# Low Molecular Weight Heparin Ablates Lung Cancer Cisplatin-Resistance by Inducing Proteasome-Mediated ABCG2 Protein Degradation

**DOI:** 10.1371/journal.pone.0041035

**Published:** 2012-07-23

**Authors:** Qi Niu, Wei Wang, Yong Li, Douglas M. Ruden, Fenghua Wang, Yue Li, Fangying Wang, Jingying Song, Kun Zheng

**Affiliations:** 1 Department of Medical Oncology, No. 309 PLA Hospital, Beijing, PR China; 2 Department of Obstetrics and Gynecology, Institute of Environmental Health Sciences, C. S. Mott Center for Human Health and Development, Detroit, Michigan, United States of America; 3 Department of Pathology, No. 309 PLA Hospital, Beijing, PR China; 4 Beijing Center for Physical and Chemical Analysis, Beijing, PR China; University Magna Graecia, Italy

## Abstract

Cancer side population (SP) cells, which are often referred to as cancer stem cells, are thought to be responsible for lung cancer chemotherapy resistance, and currently no drug can specifically target these cells. We hypothesize low-molecular-weight heparin (LMWH) may affect the biological properties of SP cells and could be used to clinically target these cells. To test this, SP cells were isolated from cisplatin (DDP)-resistant lung adenocarcinoma A549/DDP cells by flow cytometric sorting. Compared to non-SP cells, SP cells formed increased numbers of colonies *in vitro*, and had a 1000-fold increase in tumorigenicity *in vivo*. Proliferation and apoptosis assays demonstrated LMWH had no significant effect on lung SP cell proliferation or apoptosis. However, LMWH reduced lung SP cell colony formation ability and protein expression of the multidrug transporter, ABCG2, by FACS and western blot analyses without affecting its mRNA levels by RT-PCR. Consistently, immunohistochemistry stainings of ABCG2 in LMWH-treated tumor tissues were significantly reduced compared with those in controls. Further, we found proteasomal inhibitor MG132, but not lysosomal inhibitors leupeptin and pepstatin A, could restore ABCG2 protein levels in LMWH-treated SP cells. These suggest LMWH ablates lung SP cell chemoresistance by proteasome-mediated reduction of ABCG2 protein levels without affecting its mRNA levels. We also determined LMWH combined with cisplatin could overcome cisplatin-resistance and induced lung SP cells apoptosis both *in vitro* and *in vivo*. This study provides an experimental basis for using a combination of LMWH, which targets lung SP cells, with chemotherapy to improve lung cancer survival.

## Introduction

Lung cancer is the most common cause of cancer death worldwide. Currently, lung cancer survival rates are poor, with a 5-year survival rate of 15% [1].

The cancer stem cell (CSC) theory states that tumors are organized in a hierarchical manner similar to normal tissues, with a sub-population of tumorigenic stem-like cells that are chemoresistant and generate the differentiated tumor cells [2]. Side population (SP) cells with CSC-like properties were first identified in leukemia and subsequently isolated from solid tumors, including breast, brain, lung, liver, prostate, colon, pancreatic, and head and neck cancers [3]–[9]. SP cells express high levels of ATP binding cassette (ABC) multidrug transporter proteins which are believed to be the basis of chemotherapy resistance and treatment failure. Breast cancer resistance protein (BRCP/ABCG2), a member of ABC proteins family, is a major drug transporter which is considered to play a key role in protecting SP cells from cytotoxic agents, such as cisplatin, and is involved in multidrug resistance [10]–[12].

Despite the fact many agents which target CSC cells have been identified, these potential agents are far from use in the clinic. Candidate CSC cell targeting molecules affect Wnt, EpCAM, Hedgehog, and Delta/Notch signaling, while other approaches including anti-Delta-like 4 ligand (DLL4) antibody, salinomycin, metformin, TGFβ, sulforaphane, and others have been tested to modify CSC drug-resistance properties [13]–[17]. Although many of these agents appear promising, particularly *in vitro*, the major problem of specific targeting to CSCs cells and avoidance of *in vivo* toxicity remains.

Low molecular weight heparin (LMWH) was approved by the FDA in 1998 for anticoagulant therapy and has been administrated safely for over 13 years [18]. Studies showed that low molecular weight heparin treatment reduced cancer mortality in patients with deep vein thrombosis in various cancers, which is not related to the differences in death rates of thromboembolism [19]–[21]. Although several clinical studies have shown that LMWH reduced mortality and prolonged survival in patients with advanced solid malignancy, the mechanism through which LMWH exerts an anticancer effect remains undefined [22]–[25].

We hypothesize LMWH may be able to modify the biological properties of lung SP cells with CSC-like properties. Therefore, this study investigates whether LMWH affects lung SP cell proliferation, apoptosis, and chemoresistance as well as tumor growth *in vivo* and the mechanism of which LMWH takes effects. Our studies suggest LMWH could be a safe lung SP cell targeting drug which could be immediately used in the clinic to overcome chemotherapy resistance and improve lung cancer survival.

## Results

### Side Population Sorting

**Figure 1:**
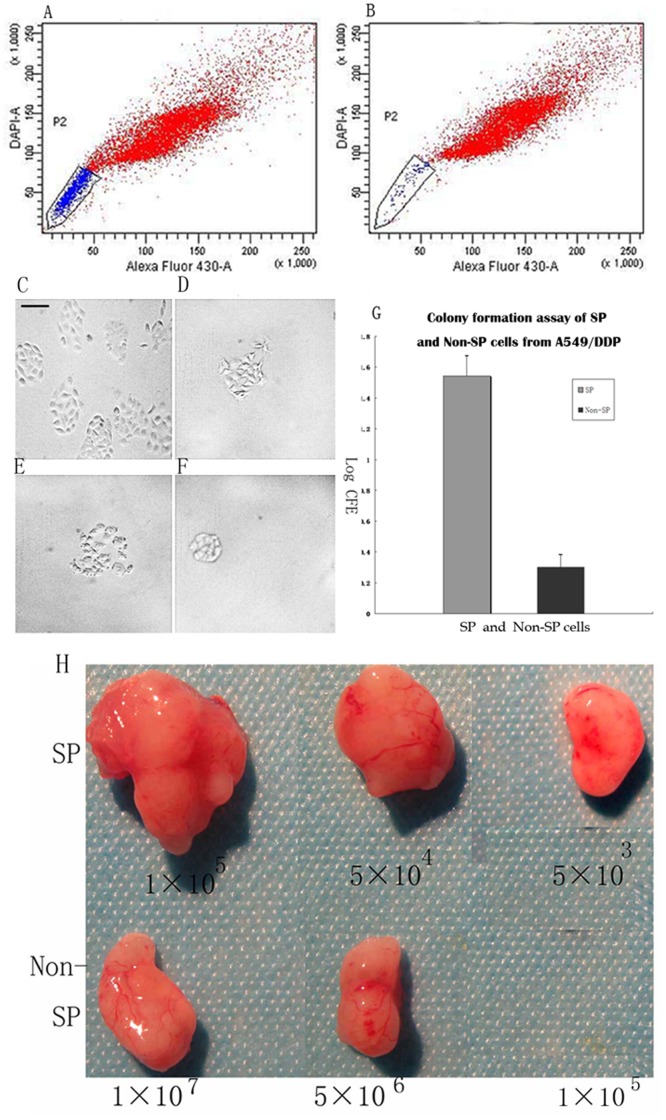
A549/DDP side population (SP) sorting and SP characters analysis. (A) A549/DDP cells incubated with Hoechst 33342, without verapamil, and the boxed region indicates the SP on FACS. (B) A549/DDP cells incubated with Hoechst 33342 in the presence of verapamil, a control to identify the SP region on FACS. Representative micrographs of A549/DDP colonies formed after 10 days from (C) SP cells and (D) non-SP cells grown in serum-free medium and (E) SP cells (F) non-SP cells treated with LMWH, scale bar = 50 µm. (G) Log colony forming efficiencies (CFE) of SP and non-SP cells grown in serum-free medium, *p*<0.05. (H) Tumors formed after inoculation of different numbers of A549/DDP side population (SP) and non-SP cells in NOD/SCID mice after 14 days.

Flow cytometry analysis with Hoechst33342 staining demonstrated that a SP of 10%–17% cells existed in A549/DDP cells. As a positive control, the numbers of SP cells were diminished in the presence of Verapamil, a drug known to inhibit the ABC trasporter pump responsible for the side population phenotype. The purity of SP cells was >95% (average 97%) and the purity of non-SP cells was 95%. SP and non-SP A549/DDP cells were sorted separately and used for further experiments (Figure 1).

### LMWH Decreases SP Cell Colony Formation Ability

The ability of SP and non-SP cells to form colonies was tested *in vitro*. Colonies of both cell types were visible after 10 days; however, the number of colonies was significantly increased in SP cells compared to non-SP cells. The Log10 colony forming efficiency (CFE) of SP cells was significantly higher than non-SP cells (1.544±0.150 vs 0.301±0.082, *p*<0.05, Figure 1). LMWH significantly reduced CFE in SP cells (1.544±0.150 vs. 0.812±0.082, *p*<0.05) compared with non-SP cells (0.301±0.082 vs 0.295±0.053, p>0.05).

### SP Cells have Increased *in vivo* Tumorigenicity

The *in vivo* tumorigenic assay indicated that 5×10^3^ SP cells were sufficient for tumor formation *in vivo*, whereas more than 5×10^6^ non-SP cells were needed to initiate tumors (Figure 1). Tumors were found in all six mice injected with 5×10^5^ and 5×10^4^ SP cells, but no tumor was found in mice injected with the same number of non-SP cells. No tumors were observed when 1×10^6^ non-SP cells were inoculated. We conclude that there was a greater than 1,000-fold difference in tumorigenicity between SP and non-SP cells.

### LMWH does not Affect SP Cell Proliferation

**Figure 2:**
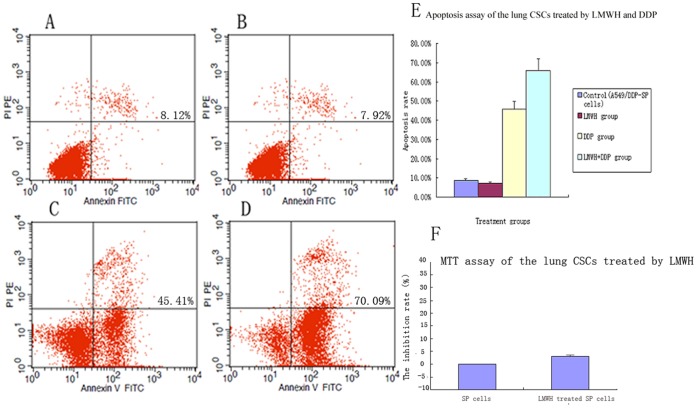
Apoptosis assay by FACS and MTT assay in A549/DDP SP cells treated with LMWH. Annexin V staining in (A) control SP cells (B) SP cells treated with LMWH (C) SP cells treated with cisplatin and (D) SP cells treated with LMWH plus cisplatin. (E) Quantification of apoptosis rates in A549/DDP SP cells induced by LMWH and DDP. No significant difference was observed in control and LMWH-treated SP cells. Apoptosis induced by LMWH plus DDP was significantly higher than DDP alone (*p*<0.05). (F) The inhibition rate of LMWH-treated A549/DDP SP cells in MTT assay. No significant difference was observed in inhibition rate of control and LMWH-treated SP cells.

The effect of LMWH on SP cell proliferation was determined using the MTT assay. LMWH had no significant effect on SP cell proliferation compared with the control SP cells (the inhibition rate of LMWH-treated SP cells was 3.11% ±0.20%, *p*>0.05,Figure 2).

### LMWH Increases Cisplatin (DDP) Induced Apoptosis in SP Cells

The effect of LMWH on apoptosis in SP cells was analyzed by FACS. There was no significant difference in the rate of apoptosis in LMWH treated SP cells and control SP cells (7.21% ±0.81% vs. 8.01% ±0.91%, *p*>0.05); however, the rate of apoptosis in the LMWH plus DDP treated SP cells (65.89% ±5.98%) was significantly higher than SP cells treated with DDP alone (45.74% ±4.12%, *p*<0.05) and control SP cells (8.01% ±0.91%, *p*<0.05, Figure 2).

### LMWH Decreases Expression of the SP Cell Surface Marker ABCG2

**Figure 3:**
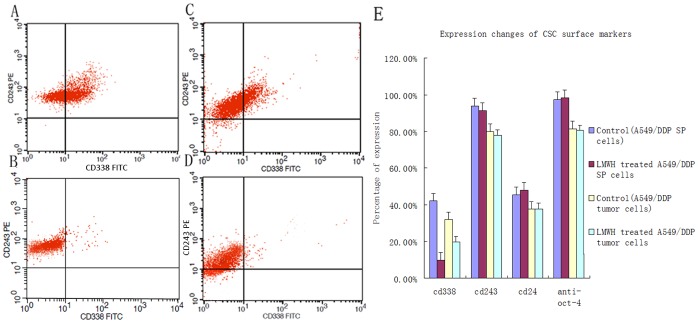
Flow-cytometry analysis of CSC marker expression induced by LMWH in A549/DDP side population (SP) cells or in A549/DDP SP xenograft tumor cells. (A) Expression of ABCG2 (CD338) in control SP cells and (B) induced by LMWH in A549/DDP SP cells. (C) Expression of ABCG2 (CD338) in controls tumor cells and (D) induced by LMWH in A549/DDP SP xenograft tumor cells. (E) Quantification of expression of CSC marker expression induced by LMWH in SP cells and xenograft tumor cells. LMWH significantly downregulated expression of ABCG2 by FACS (*p*<0.05).

A549/DDP SP cells express ABCG2, CD243 (p-gp) and CD24 at high levels. After incubation with LMWH for 36 h, ABCG2 protein expression was significantly reduced in SP cells: ABCG2 expressing cells decreased from 42.25% ±4.11% to 9.92% ±4.08%, (*p*<0.05, Figure 3). No significant change was observed in the expression of p-gp and Oct-4 after incubation with LMWH (Figure 3).

### FACS Analysis of ABCG2 Expression in Tumor Cells

Cells obtained from the xenograft tumor tissues expressed ABCG2, CD243 (p-gp) and CD24. The protein expression of ABCG2 in tumor tissues of LMWH treated mice were significantly lower than those in control mice (30.38% ±2.13% vs 20.22% ±2.01%, *p*<0.05, Figure 3). No significant change was observed in the expression of p-gp and Oct-4 after incubation with LMWH (Figure 3).

### LMWH does not Affect ABCG2 mRNA Expression in SP Cells and Tumor Cells with RT-PCR

**Figure 4:**
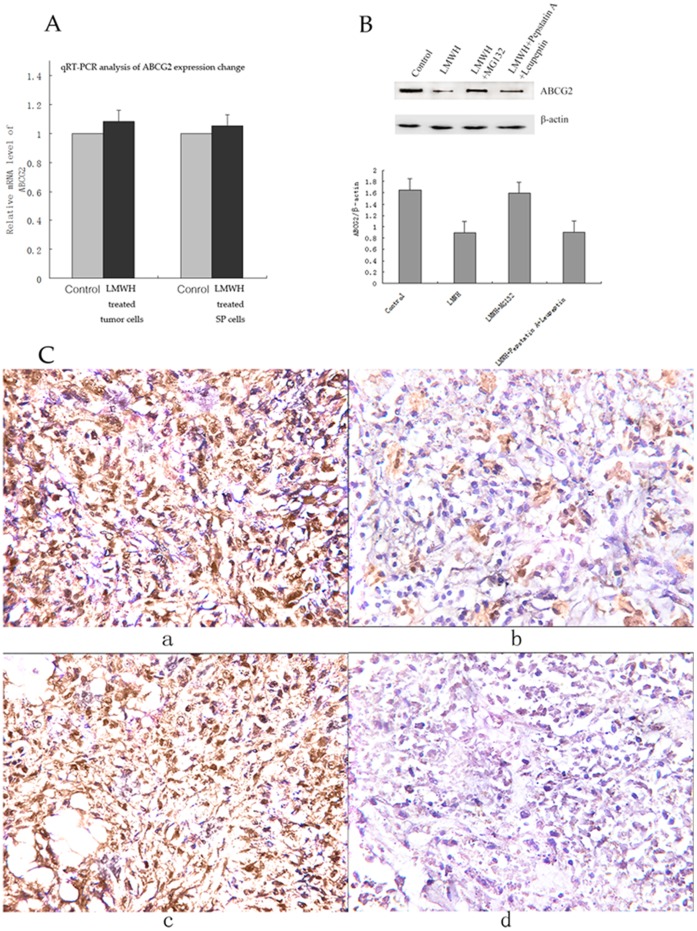
ABCG2 expression analysis with quantitative real time RT-PCR, western blot and immunohistochemistry staining. (A) ABCG2 mRNA expression changes in LMWH treated tumor cells and LMWH treated SP cells. Quantitative real time RT-PCR shows that the expressions of ABCG2 mRNA were not significantly changed in LMWH treated tumor cells or LMWH treated SP cells compared with those in controls (*p*>0.05). β-actin was used as an internal reference. (B) Western blot analysis of ABCG2 expressions in SP cells treated with LMWH, LMWH+MG123, LMWH+Pepstatin A+Leupeptin, and control. SP cells were initially treated with LMWH or control for 10 hours, and then MG132 (10 µM), Pepstain A + Leupeptin (each 100 µg/ml), or control were added correspondingly and incubated for 6 hours. Then cells were harvested for western blot analysis. β-actin was used as a loading control. The densities of the ABCG2 protein bands in western blot were quantified with the Quantity One-4.6.2 program. (C) ABCG2 expressions in LMWH treated and control tumor tissues (IHC, SP×200). The average SI score of ABCG2 expression in LMWH treated tumor tissues was 3.63 compared with the average SI score of 9.13 in control tumor tissues (3.63±1.69 vs 9.13±2.03, *p*<0.05).

To determine if LMWH affets ABCG2 expression at the mRNA level, we performed qRT-PCR analysis of SP cells treated with LMWH for 16 hours and tumor cells from LMWH treated and control mice. No significant change was found in ABCG2 mRNA level following LMWH treatment (Figure 4). Thus, LMWH unlikely affects ABCG2 expression at its mRNA level.

### ABCG2 Protein Expression can be Restored by the Proteasomal Inhibitor MG132 in LMWH-treated SP Cells

Since we did not find significant change of ABCG2 mRNA levels in SP and tumor cells, we tested if LMWH induced ABCG2 reduction is caused by ubiquitin-mediated protolysis or by the lysosome. To test this, SP cells were treated with LMWH, LMWH + MG132, and LMWH + Pepstatin A + Leupeptin. As shown in Figure. 4, ABCG2 protein expression in LMWH treated SP cells was significantly reduced compared with that in control after 16 hours treatment. The reduction could be restored by proteasomal inhibitor MG132 treatment while lysosomal inhibitors pepstatin A plus leupeptin had no effect. These suggest that LMWH induced ABCG2 protein degradation through the proteasome pathway.

### LMWH Treatment Decreases ABCG2 Protein Expression in Tumor Tissue by Immunohistochemistry

ABCG2 expression was present in different intensities and different cell distributions by immunohistochemistry staining. Significantly low level of expression of ABCG2 was observed in LMWH treated tumor tissues compared with controls. Following the staging criteria of stain intensity (SI), the average SI score of ABCG2 expression in LMWH treated tumor tissues was 3.63 compared with the average SI score of 9.13 in control tumor tissues (3.63±1.69 vs 9.13±2.03, *p*<0.05, Figure 4).

### Cisplatin Xenograft Growth Delay is Increased by LMWH

**Figure 5:**
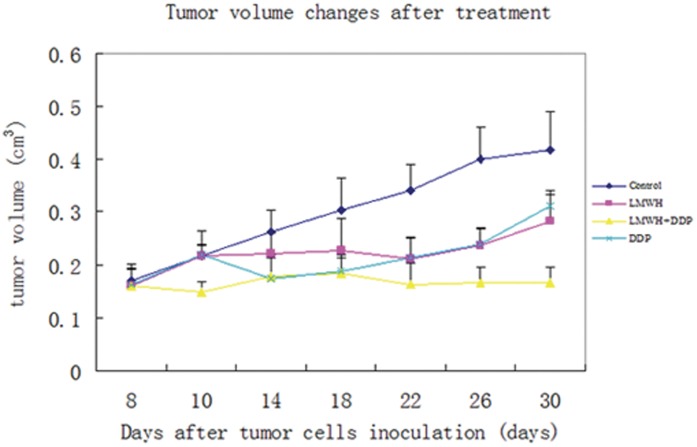
A549/DDP side population (SP) tumor xenograft growth. A549/DDP SP cells were inoculated in Balb/C nude mice and tumor volumes were recorded beginning day 1 of treatment with normal saline control, LMWH, DDP and DPP plus LWMH. The differences of tumor sizes in LMWH combined with cisplatin and cisplatin-treated tumors was significant after 25 days (0.17±0.03 cm^3^ vs 0.24±0.08 cm^3^, *p*<0.05) and within 23 days in control animals (0.16±0.09 cm^3^ vs 0.27±0.09 cm^3^, *p*<0.05). No significant difference in tumor volume of the LMWH and DDP groups was found from day 8–30.

Administration of LWMH plus DDP to A549/DDP tumor-bearing mice resulted in significantly reduced tumor growth compared with DDP alone (0.17±0.05 cm^3^ vs 0.29±0.06 cm^3^, *p*<0.05) or control mice (0.44±0.09 cm^3^, *p*<0.05) after 30 days. In A549/DDP tumor-bearing nude mice, the differences in LMWH combined with cisplatin and cisplatin-treated tumors was significant after 25 days (0.17±0.03 cm^3^ vs 0.24±0.08 cm^3^, *p*<0.05) and within 23 days in control animals (0.16±0.09 cm^3^ vs 0.27±0.09 cm^3^, *p*<0.05, Figure 5). No significant difference in tumor volume of the LMWH and DDP groups was found from day 8–30 (Figure 5).

## Discussion

Isolation of lung SP cells with CSC-like properties and identification of existing drugs which modify lung SP cells and overcome their chemoresistance may provide innovative strategies to improve clinical outcome in lung cancer. We demonstrated that SP cells from A549/DDP cisplatin-resistant human lung adenocarcinoma cells are enriched in CSC-like properties as they have significantly increased colony formation *in vitro* and tumorigenicity *in vivo*, indicating that A549/DPP SP cells provide a good model to study lung SP cells and a novel system to study chemoresistance.

Our study indicates that LMWH does not directly kill lung SP cells or induce SP cell apoptosis *in vitro*. However, LMWH sensitizes cisplatin-resistant SP cells to cisplatin induced apoptosis *in vitro*. *In vivo* experiments demonstrate that LMWH combined with cisplatin significantly inhibits cisplatin-resistant tumor xenograft growth. Further studies demonstrate that LMWH significantly reduces ABCG2 protein expression, by FACS and western blot analyses. Yoh found that expression of the ABCG2, but not of Pgp, MRP1, MRP2, and MRP3, was a predictive factor of poor clinical outcome in advanced NSCLC patients even though the role of ABCG2 as transporter of platinum conjugates is still controversial [12]. Our results are consistent with his findings and all these clearly suggest ABCG2 is a drug transporter that plays a major role in cisplatin resistance in SP cells. Immunohistochemistry analyses consistently showed that LMWH significantly reduces ABCG2 protein expression in LMWH-treated tumor tissues compared with controls. All these suggest that LMWH can prevent chemoresistance of SP cells by reducing ABCG2 protein expression without affecting its mRNA levels.

Further studies reveal that the ABCG2 protein reduction in SP cells was due to induced degradation by LMWH through the ubiquitin-proteasome pathway because proteasomal inhibitor-MG132, but not lysosomal inhibitors- leupeptin and pepstatin A, restores ABCG2 protein expression in LMWH-treated SP cells. We speculate that LMWH might bind to ABCG2 in SP cell membranes and induce ubiquitinization and degradation, which reduces chemoresistance of lung SP cells.

LMWH is a safe anticoagulant agent used in coagulant and non-coagulant conditions, which has been used in cancer treatment for more than a decade [18]. Retrospective data suggests that it could improve survival in some cancer patients, but the exact mechanism remains unclear [26]. Experimental models suggest LMWH is able to inhibit tumor growth, invasion, metastasis, angiogenesis, matrix formation and reverse multidrug resistance [27]–[31]. This study shows that LMWH alone could moderately reduce tumor growth *in vivo* even though it could not induce tumor cells apoptosis directly *in vitro*. The reason could be that LMWH exerts its tumor inhibitory effect through interference with tumor cell adherence, invasion, angiogenesis, or microenvironement [32], [33].

To our knowledge, this study is the first report that LMWH can prevent chemoresistance of lung SP cells by reducing ABCG2 protein expressions through proteasome-dependent degradation. The ability of LMWH to sensitize SP cells to chemotherapy by inducing ABCG2 degradation through the proteasome pathway may provide an explanation for the unknown mechanism by which LMWH improves chemotherapy response and survival in cancer patients [28], [34].

In conclusion, our studies suggest that LMWH reduces lung cancer chemoresistance via inducing ABCG2 protein degradation through the proteasome pathway. The combination of LMWH with cisplatin could target both chemoresistant lung SP cells and chemosensitive non-SP cancer cells, providing an effective new strategy for lung cancer treatment. As LMWH can be used independently of hypercoagulant conditions and the side effects are well tolerated, the clinical application of LMWH in lung cancer has great potential. Clearly, further clinical studies of LMWH combined with chemotherapy and radiotherapy in lung cancer are needed, and it will be worthwhile to explore whether LMWH can modify SP cells from other cancer types.

## Materials and Methods

### Cancer Cell Culture and Reagents

The cisplatin-resistant human lung adenocarcinoma cell line A549/DDP was obtained from the American Type Culture Collection (Rockville, MD, USA) and routinely cultured in RPMI medium supplemented with 10% human AB serum and 2 µmol cisplatin (Qilu Pharmaceutical Co, Ltd, Shandong, China). A549/DDP cells were cultured in complete RPMI medium without cisplatin for 3 days before being used in experiments. Leupeptin, pepstatin A, and MG132 were bought from Sigma (St Louis, MO, USA).

### Side Population Sorting and Analysis

A549/DDP cells were suspended at 1×10^6^ cells/ml in PBS containing 4% FBS, incubated at 37°C for 60 min with 5 µg/ml Hoechst 33342 (Sigma, St Louis, MO, USA) either alone or in the presence of 500 µmol verapamil (Sigma, St Louis, MO, USA). After incubation, 1 µg/ml propidium iodide was added and then the suspension was filtered through a 40-µm cell strainer (BD Bioscience, San Diego, CA, USA) to obtain a single cell suspension. Flow cytometery analysis and sorting were performed using a FACS Vantage SE (BD Bioscience). Hoechst 33342 was excited with the UV laser at 350 nm and fluorescence emission was measured with 405/BP30 (Hoechst blue) and 570/BP20 (Hoechst red) optical filters. The purity of SP cells and non-SP cells was tested again using FACS. After FACS sorting, SP and non-SP A549/DDP cells have been kept in complete RPMI medium, and were used for further experiments within 2 hours.

### Colony Formation Assay

Colony formation assay was performed to assess the clonogenic ability of SP and non-SP cells. After sorting, 100 SP or 100 non-SP A549/DDP cells were plated in RPMI 1640/10% on 6-well plates in triplicate. The media was changed twice a week and after 10 days, the number of colonies in each well was determined under a microscope and representative fields were photographed. Percent colony forming efficiency (%CFE) was calculated as = (Number of colonies obtained/Number of cultured cells)×100 and the log transformed values were calculated [35], [36].

### *In vivo* Tumorigenicity Experiment

All animal experiments were conducted with the approval of the Institutional Animal Care and Use Committee of the 304 PLA Hospital Institute for Biological Sciences. Twenty four five-week old non-obese diabetic/severe combined immunodeficiency (NOD/SCID) male mice were purchased from the Chinese Association for Laboratory Animal Science (CALAS; Beijing, China) and housed under specific pathogen-free conditions, in a controlled temperature and humidity environment with 12 h light/dark cycles. Mice were injected subcutaneously on one side with 5×10^5^, 1×10^5^, 5×10^4^ or 5×10^3^ SP cells or 1×10^7^, 5×10^6^, 5×10^5^ or 5×10^4^ non-SP cells in 100 µl suspensions and tumor growth was monitored. 0.3 mg/0.20 ml LMWH was injected subcutaneously in LMWH treatment group at day -1. Mice were sacrificed at day 60 or when tumors reached a maximum volume of 1,000 mm^3^. Tumor volume was calculated using (width^2^×length)/2. Fold difference in tumorigenicity was calculated as minimum number of non-SP cells needed to generate a tumor/minimum number of SP cells needed to generate a tumor.

### MTT Assay

A549/DDP SP sorted cells were seeded into 96-well plates at 7.5×10^3^ per well and treated with or without 5 IU/ml LMWH (Low-molecular weight heparin calcium; Bopuqin, TianJing Chase Sun Pharmaceutical Co, Ltd, TianJing, China) in triplicate. Three wells containing tumor cells suspended in complete medium were used as controls for cell viability and three wells containing only complete medium were used as controls for nonspecific dye reduction. After 48 hours, MTT dye solution was added to each well and samples were incubated at 37°C for 4 h, the formazan product was dissolved by adding 100 µl DMSO to each well and absorbance (A) were read at 570 nm using an ELISA Reader (Anthos 2020, Salzbrug, Australia) The inhibition rate (%) was calculated as 100%×(control group A values –experimental group A values)/control group A values.

### Apoptosis Assay and SP Cell Marker Expression Analysis

A549/DDP SP cells (1×10^6^/96 well plate) were untreated (control), or treated with 5 IU/ml LMWH, LMWH plus 2.2 µg/ml cisplatin (DDP), or DDP alone for 36 h and stained using the Annexin V-FITC apoptosis detection kit (BioVision, Mountain View, CA, USA) and analyzed on a FACScalibur flow cytometer (BD Bioscience). For SP cell marker analysis, A549/DDP SP cells were incubated with LMWH for 36 h, then incubated for 30 min at 4°C with monoclonal antibodies against p-gp (Abcam, Hong Kong Science Park, New Territories, Hong Kong), ABCG2 (Abcam, Hong Kong Science Park, New Territories, Hong Kong), CD24 (Biolegend, Roselle Street, San Diego, CA, USA), and Oct-4 (Biolegend, Roselle Street, San Diego, CA, USA) coupled to FITC or PE (Biolegend, San Diego, CA, USA) at the concentration recommended by the manufacturer and analyzed by flow cytometry. Apoptosis assay and SP cell marker expression analysis were repeated 3 times.

### Quantitative Real-time RT-PCR

A549/DDP SP cells of LMWH treated or untreated and tumor cells collected from LMWH treated or untreated mice were suspended at 1×10^6^ cells/ml in 5 ml PBS as sample respectively. Then, isolation of total RNA was performed using TriPure Isolation reagent (Roche Diagnostics GmbH. Germany) or RNeasy Mini Kit (Qiagen, USA) according to manufacturer’s instructions.

The mRNA levels were analyzed by quantitative real time RT-PCR using a Bio-Rad iCycler system (Bio-Rad, Hercules, CA). The mRNAs were reverse-transcribed into cDNAs by using an iScript cDNA synthesis kit (Bio-Rad, Hercules, CA). The primer sequences used in real-time RT-PCR are: 5′-GGGTAATCCCCAGGCCTCTA-3′ (sense primer of human ABCG2 gene), 5′-CCAGCTCTGTTCTGGATTCCA-3′(antisense primer of human ABCG2 gene), 5′-AGCGAGCATCCCCCAAAGTT-3′ (sense primer of human β-actin gene), and 5′-GGGCACGAAGGCTCATCATT-3′(antisense primer of human β-actin gene). The reaction was performed under the following conditions: one cycle at 48°C for 45 min; 95°C for 2 min; 40 cycles at 95°C for 30 s, 55°C for 1 min, and 68°C for 2 min. Each RT-PCR amplification was repeated in triplicate. The PCR efficiency was examined by serially diluting the template cDNA and the melting curve data were collected to check the PCR specificity. Each cDNA sample was triplicated and the corresponding no-RT mRNA sample was included as a negative control. The β-actin primer was included in every plate to avoid sample variations. The mRNA level of each sample was normalized to that of the β-actin mRNA. Relative mRNA level was presented as 2^-[(Ct/ABCG2- Ct/β-actin )LMWH -(Ct/ABCG2- Ct/β-actin)control]^. All data shown were the mean±SD of three separate experiments.

### Tumor Growth Xenograft Study

Six-week-old inbred male Balb/C nude mice were obtained from the Institute of Chinese Association for Laboratory Animal Science (CALAS). A549/DDP lung adenocarcinoma cells (5×10^6^) were subcutaneously inoculated in the upper left flank on day 1. When the diameters of tumors were >5 mm, the mice were divided into control, DDP, and LMWH with DDP groups (n = 10 each) and treated with i.p. injection of 8 mg/kg DDP or an equal volume of saline solution once a week for 3 weeks. LMWH (0.3 mg/0.20 ml) or saline solution was given s.c. every day from day 8 to day 30. Tumor volume was determined three times per week as previously described and mice were euthanized on day 30.

### SP Cell Marker Expression in Tumor Explants

Tumor samples were excised from mice, rinsed, mechanically minced, and digested for 4 h at 37°C in a shaking incubator with 0.1 Wünsch units/ml collagenase I (Roche Diagnostics) in DMEM. The digest was disaggregated using an 18.5-gauge needle, sieved through a 100 µm cell strainer and prepared for flow cytometry analysis of SP cell marker expression as described previously.

### Western Blot

SP cells were initially treated with LMWH or control for 10 hours, and then MG132 (10 µM), Pepstain A+ Leupeptin (each 100 µg/ml), or control were added correspondingly and incubated for 6 hours. Then cells were harvested for western blot analysis. Primary antibody incubation was carried out at 4°C for 16 hours with a mouse anti-ABCG2 antibody (Biolegend, San Diego, CA, USA) diluted at 1∶300 in 3% bovine serum albumin, followed by incubation with horseradishperoxidase–conjugated horse anti-mouse secondary antibody, and developed using an enhanced chemiluminescence detection system (GE Healthcare, Piscataway, NJ, USA). Anti–β-actin antibody was used as the loading control (Santa Cruz Biotechnology, Santa Cruz, CA, USA).

### Immunohistochemistry Staining of ABCG2 in Tumor Tissue

The paraffin-embedded tissues were cut into 4 µm slides and baked at 60°C for 60 min. The sections were de-paraffinized with xylenes and rehydrated. Then sections were submerged into EDTA antigenic retrieval buffer in a pressure cooker for 10 minutes and then cooled at room temperature for 20 minutes. The sections were treated with 3% hydrogen peroxide in methanol to quench the endogenous peroxidase activity, followed by incubation with normal serum to block nonspecific binding. Then, the slides were incubated with Purified anti-human ABCG2 antibody (Abcam, Hong Kong Science Park, New Territories, Hong Kong, China) with a dilution of 1∶20 at 4°C overnight. The second antibody was from an SP reagent kit (Zhongshan Biotechnology Company, Beijing, China). After washing, the tissue sections were treated with biotinylated anti-mouse secondary antibody, followed by further incubation with streptavidin-horseradish peroxidase complex for 20 mins. Stained with diaminobenzidine (DAB), the sections were counterstained with hematoxylin. The known positive tissue was used for positive controls. The primary antibody was omitted for negative controls. An Olympus (Olympus,Tokyo, Japan) microscope was employed to visualize the staining of targeted proteins.

The stained slides were reviewed and scored independently by two observers and the scores were determined by combining the proportion of positively stained tumor cells and the intensity of staining. Tumor cell proportion was scored as follows: 0 (no positive tumor cells); 1 (≤30% positive tumor cells); 2 (31–50% positive tumor cells); 3 (51–80% positive tumor cells) and 4 (>80% positive tumor cells). Staining intensity was graded according to the following criteria: 0 (-, no staining); 1 (+, weak staining = light yellow); 2 (++, moderate staining = yellow brown) and 3 (+++, strong staining = brown). Staining index (SI) was calculated as the product of the staining intensity score and the proportion of positive tumor cells. Using this method of assessment, we evaluated ABCG2 expression in tumor tissues by determining the SI, with scores of 0, 1, 2, 3, 4, 6, 8, 9 or 12.

### Statistical Analysis

Data are presented as mean ± S.D. Paired and unpaired student’s *t*-test were used to analyzing two groups of paired or unpaired data, respectively. Repeated measured analysis of variance (ANOVA) was used for comparison of multiple groups. Differences were considered significant at *p*<0.05.
